# The validity of the upper limb neurodynamic test 2A in women with a clinical diagnosis of carpal tunnel syndrome: a prospective diagnostic accuracy study

**DOI:** 10.11604/pamj.2022.42.61.30119

**Published:** 2022-05-23

**Authors:** Hassan Beddaa, Bouchra Kably, Ikrame Mouhi, Basma Marzouk, Abdelghafour Marfak, Said Nafai, Reda Ouazzani, Nazha Birouk

**Affiliations:** 1 Clinical Research Biostatistics and Epidemiology Laboratory, Faculty of Medicine and Pharmacy, Mohamed V University of Rabat, Rabat, Morocco,; 2 Clinical Neurophysiology Department, Specialty Hospital, Ibn Sina University Hospital Center, Mohamed V University of Rabat, Rabat, Morocco,; 3 National School of Public Health, Rabat, Morocco,; 4 Division of Occupational Therapy, School of Health Sciences, American International College, USA

**Keywords:** CTS, neurodynamic test, sensitivity, specificity, mechanosensitivity, manual therapy, physiotherapy

## Abstract

**Introduction:**

the validity of the upper limb neurodynamic tests and especially the upper limb neurodynamic test 1 for diagnosing carpal tunnel syndrome has been the subject of several previous studies. However, the upper limb neurodynamic test 2A, which is also a test designated to assess the mechanosensitivity of the median nerve, has not been sufficiently studied, particularly for the diagnosis of carpal tunnel syndrome.

**Methods:**

we used the upper limb neurodynamic test 2A as the index test and nerve conduction studies as the reference standard. We considered the upper limb neurodynamic test 2A positive according to Nee et al. criteria. Sensitivity, specificity, positive likelihood, and negative likelihood were calculated. In addition, a receiver operating characteristics analysis was carried out.

**Results:**

ninety-four women (188 hands) suspected of carpal tunnel syndrome with a mean age of 48.87 years and SD of 12.09 participated in the study. The sensitivity of the upper limb neurodynamic test 2A was estimated at 73.4%, the specificity at 47%, the positive likelihood ratio was 1.38, the negative likelihood ratio was 0.57, and the Kappa agreement was 20.3%, and the area under the curve 60.1%.

**Conclusion:**

the upper limb neurodynamic test 2A does not seem to have value in the diagnosis of carpal tunnel syndrome when compared to nerve conduction studies. It could be alternatively used to detect an increased mechanosensitivity of the median nerve when the upper limb neurodynamic test 1 cannot be performed in case of a range of motion limitation of the shoulder abduction.

## Introduction

Carpal tunnel syndrome (CTS), a localized compression of the median nerve at the wrist, consists of a combination of symptoms such as hand pain, numbness, tingling, and burning in the distal distribution of the median nerve [1]. It is considered the most common nerve entrapment affecting the upper limb. Its prevalence was estimated at 3.8% in the general population, whereas 5.8% was confirmed by NCS [2,3]. Several studies have shown that it is more frequent in the female population and associated with repetitive handwork and extreme wrist posture [4,5]. Functional limitations regarding daily activities, absenteeism from work, and the cost of managing this condition are parts of the socio-economic impact of this disorder [6,7]. The diagnosis of CTS is based on history, physical examination, ultrasound, and Nerve Conduction Studies (NCS) [8,9]. NCS are still considered the reference standard for diagnosing CTS because of its high sensitivity and specificity [8]. In addition, many studies were conducted to explore the value of ultrasound in diagnosing CTS. The results showed its usefulness in the case of negative NCS findings in patients with CTS clinical presentation [10,11].

Furthermore, several median nerve provocation tests such as Phalen, Reverse Phalen, and Tinel are commonly used to highlight CTS symptoms. They have shown conflicting results for the diagnosis of this condition, though [12]. Because of this, several previous studies tried to evaluate the validity of the Upper Limb Neurodynamic Tests (ULNTs) and particularly ULNT1 for the diagnosis of CTS. Indeed, ULNTs were recommended for the diagnosis of CTS as well as other neuropathic pain conditions in departments and areas with limited access to NCS [13]. Clinicians use neurodynamic tests to determine peripheral nerve disorders. They are designed to put mechanical stress on the neural tissue and assess its mechanosensitivity changes, which refers to the pain resulting from neural structures during their mobility or posture [14]. Consequently, positive and negative signs and symptoms might occur during a neurodynamic test showing abnormal excitability of the nervous system, as demonstrated by Jaberzadeh *et al*. in 2013 for ULNT1 in patients with chronic CTS.

Many studies have tried to assess the accuracy of ULNTs for the diagnosis of CTS to expand the range of used tests [15-18]. They have indeed shown results encouraging their use. However, the majority of these studies were only interested in ULNT1. Moreover, the performance of this test requires a sufficient Range Of Motion (ROM) of the different joints crossed by the nerve and especially a shoulder abduction of 110°[19,20]. Despite its clinical relevance, the test cannot be performed when this mobility cannot be achieved. That is why ULNT2A, which is also a neurodynamic test for the median nerve that only requires an abduction of the shoulder of 30° to 40° [21], can represent a good alternative when impossible to carry out ULNT1 because of a decreasing ROM of the shoulder joint. This study aims to determine the validity of the ULNT2A in women with a clinical diagnosis of CTS.

## Methods

**Ethical considerations:** the study was authorized by the Biomedical Research Ethics Committee at the Faculty of Medicine and Pharmacy of Rabat, part of Mohammed V University, on December 20^th^, 2018 (Decision No. 13/19). All participants were informed about the study aims and that they could withdraw at any time without giving any reason. All the participants gave their written consent to participate in the study.

**Study design:** a prospective diagnostic accuracy study. The data collection was planned previously. The ULNT2A was used as the index test and the NCS as the reference standard. This study lasted 12 months between November 2019 and November 2020.

**Examiners:** the team was composed of four examiners, two physicians (history and clinical examination), one physiotherapist (ULNT2A, ROM of the shoulder and neck), and a neurophysiologist (NCS).

**Figure 1:**
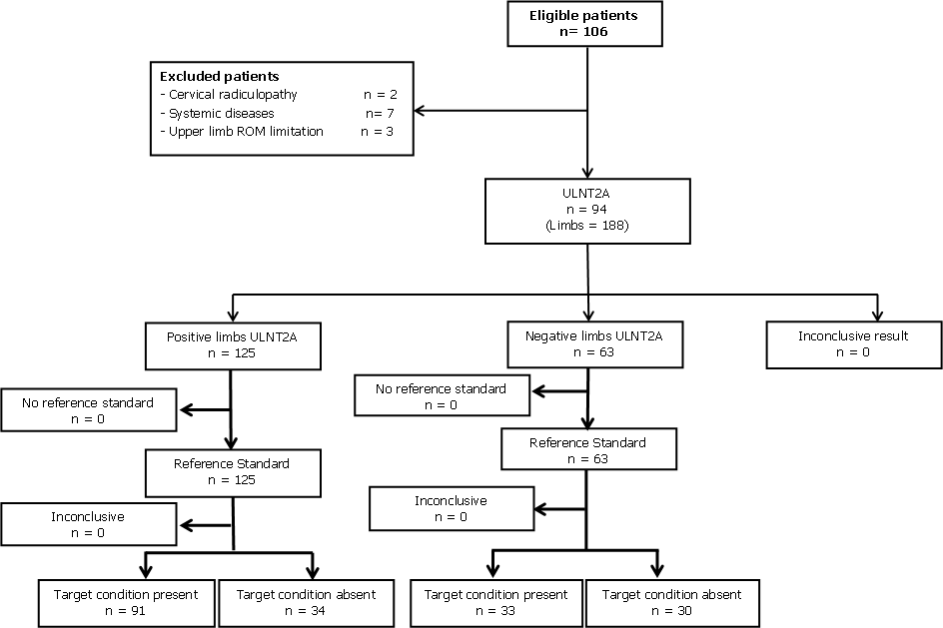
flow chart of the study according to the standards for reporting of diagnostic accuracy

**Subjects:** participants with suspected CTS were referred to the clinical neurophysiology department of Rabat Specialty Hospital (RSH), Morocco, to undergo NCS. They were voluntarily recruited at this department to participate in this study. The inclusion criteria were: patients aged > 18 years with suspected CTS and referred by their physicians for NCS. The exclusion criteria were: Age < 18 years, and any ROM limitation in the upper limbs that could limit a correct performance of the ULNT2A. Inflammatory, systemic, infectious conditions or history of fracture that may influence ULNTs. History of surgical intervention for CTS, Cervical Radiculopathy (CR), cognitive or behavioral deficits that may prevent the participants from giving correct feedback during the ULNT2A test. A flow diagram illustrating the study design according to the Standards for Reporting of Diagnostic Accuracy [22] is provided in Figure 1.

**Tests Methods:** after completing the consent form, general information (Demographic, current and previous medical and surgical history, location, occupation) was collected by a physician using a form prepared for this purpose. Another physician performed the Spurling´s and the neck distraction test to rule out participants who might have CR once symptoms could be similar to those of CTS, according to Wainner *et al*. in 2003 [23]. A physiotherapist who had 16 years of experience in physiotherapy performed passive ROM of the upper limbs and the neck to check any joint stiffness that might prevent the performance of ULNT2A. Participants were given 25 minutes of rest before performing ULNT2A to reduce median nerve sensitization after NCS. Before ULNT2A, participants have been instructed to signal the onset of symptoms by saying “stop” and how to communicate symptoms and any sensation (numbness, tingling, burning, hypoesthesia…). So, they were trained to say “worse”, “better”, or “the same” with the SD maneuver symptoms changes.

The ULNT2A was performed as described by Butler (1991). Participants were positioned diagonally in supine, the head close to the edge of the plinth, without a pillow, and the lumbar spine in a neutral position with the knees extended and the lower limb straight. The other upper limb was positioned in a neutral position. At first, the ULNT2A was carried out on the unaffected or less affected upper limb and then on the affected or more affected one until the participants´ symptoms were reproduced or until the end of the available ROM. If the symptoms were provoked, the limb movement sequence was stopped. The physiotherapist maintained this position then the SD maneuver was performed (by an active contralateral and ipsilateral lateral flexion of the participants´ neck). Each examiner was blinded to the results collected by the other examiners, and participants were also unaware of NCS findings.

**Diagnostic criteria:** the ULNT2A was considered positive, according to Nee *et al*. criteria [24] which means if participants had at least a partial reproduction of their symptoms and a positive SD maneuver (contralateral neck lateral flexion increased symptoms and ipsilateral neck lateral flexion decreased symptoms).

**Reference standard:** NCS are still the reference standard for diagnosing CTS [8]. Furthermore, since the hand´s temperature can influence the results [25], participants were asked to warm their hands with an electric heater in a room near the examination room before performing NCS. Afterward, an experienced neurophysiologist with 20 years' experience carried out NCS in a heated room, according to the protocol adopted by the clinical neurophysiology department of the RSH, which corroborated the AAEM recommendations [8].

**Statistical analysis**: the data were recorded in an electronic database, ensuring the confidentiality and anonymity of the participants. The sample characteristics and symptoms were summarized by proportion, mean and standard deviation. Statistical analysis compared positive ULNT2A with the CTS case definition to estimate the test's diagnostic accuracy. So, sensitivity, specificity, Positive Likelihood Ratio (PLR), Negative Likelihood Ratio (NLR), and their 95% confidence intervals were calculated using a two-by-two contingency table. Additionally, a receiver operating characteristics (ROC) curve analysis, a ROC curve, and an area under the curve (AUC) were carried out. All statistical analysis was performed using SPSS v.20 software.

**Funding:** the authors confirm that they have no affiliation or financial interest with any organization or entity that directly interests the subject matter or material discussed in the article.

## Results

### General characteristics

Our sample consisted initially of 106 participants, 104 women and 02 men, who were referred to the clinical neurophysiology department of the RSH between November 2019 and November 2020 for NCS. Twelve subjects were excluded from the study (2 participants because of CR, 7 participants for systemic diseases, 3 participants for ROM restriction of the upper limb). Consequently, 94 participants (188 hands) were enrolled in the study during the previously decided recruitment period. Therefore, our sample size appears reasonable compared to those identified and discussed in the current study, with a mean of 71 patients (minimum n=47 and maximum n=120 patients). Participants´ ages ranged from 18 to 78 years, with a mean of 48.87 years and SD of 12.09. The sample was composed of women (100%; 40% were housewives with many hand tasks (cleaning, baking, washing). About 31% of the employed participants were blue-collar. Chronic symptoms lasting more than three months were reported in 98% of cases (Table 1).

**Table 1:** characteristics of the sample

Characteristics of participants	Overall (n=94)
**No. females (%)**	94 (100%)
**Age (years), mean (SD)**	48.87 (12.09)
**Height (m), mean (SD)**	1.60 (0.06)
**Weight (Kg), mean (SD)**	70.67 (14.17)
**BMI (Kg/m^2^)**	27.46 (5.22)
**No. occupation (%)**	
Blue-collar	29 (31%)
White-collar	3 (3%)
Housewives´ with hand activities	38 (40%)
Not employed	24 (26%)
**No. symptoms duration (%)**	
<1 month	1 (1%)
1-3 months	1 (1%)
>3 months	92 (98%)

SD: Standard deviation; BMI: Body Mass Index

### Diagnostic accuracy of the ULNT2A

**Figure 2:**
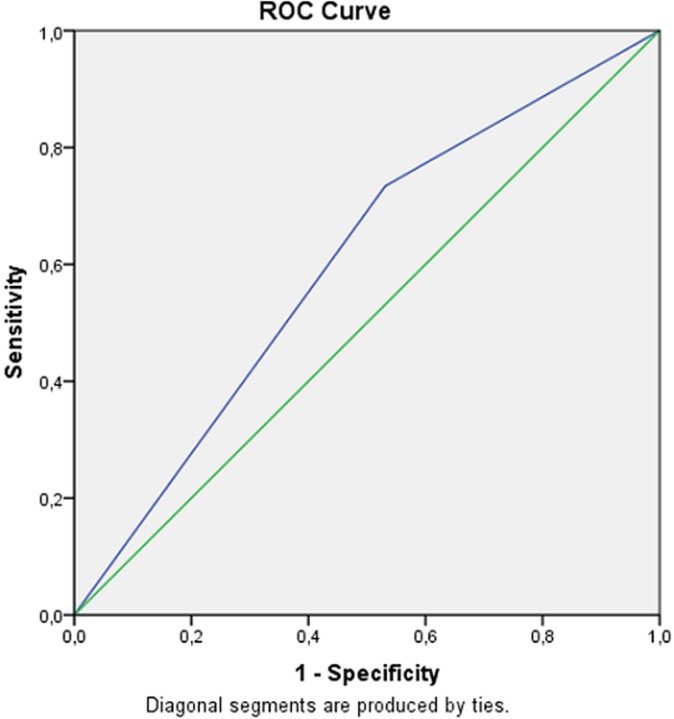
the receiver operating characteristics curve

According to Nee *et al*. criteria and NCS findings based on the case definition, the results found are illustrated in Table 2. From the 188 evaluated hands, 124 showed abnormal NCS findings. However, the ULNT2A showed 33 false-negative tests. This study estimated the sensitivity of the ULNT2A at 73.4%, 95% Confidence interval [CI]: [0.62-0.84]; the specificity at 47%; CI: [0.17-0.78], a PLR of 1.38; CI: [1.12-1.65], a NLR of 0.57; CI: [0.21-1.15], a Kappa agreement of 20.3% (P = 0.05), and an AUC of 60.1%; 95% CI: [0.51- 0.69] (P = 0.02). The ROC cure is provided in Figure 2.

**Table 2:** upper limb neurodynamic test 2A cross-tabulation results compared with nerve conduction test

Test	Nerve conduction test			
		Positive	Negative	Total
**Upper Limb Neurodynamic Test 2A**	Positive	91	34	125
	Negative	33	30	63
	Total	124	64	188

## Discussion

This study aimed to assess the validity of ULNT2A for the diagnosis of CTS in women with a clinical presentation of this disorder. The mean age of the women who took part in the study was 48.87 years, with a standard deviation of 12.09, and 98% of them had symptoms that lasted more than three months. The ULNT2A's sensitivity was estimated at 73.4%, the specificity at 47%, a PLR of 1.38, and a NLR of 0.57 in terms of test validity. Furthermore, the Kappa agreement was 20.3% which means that there is just a slight agreement between the ULNT2A and the NCS, and an AUC of 60.1%, which gives the ULNT2A a poor clinical value for diagnosing CTS.

To our knowledge, the current study is the first to assess the validity of the ULNT2A test for the diagnosis of CTS in women in the past ten years. Previous studies have all attempted to investigate the validity of the ULNT1 test in diagnosing CTS. As the two tests are designated for the median nerve with a difference in sequence and ROM [20,26], it seemed logical to compare the results of this study with the results of studies on the validity of the ULNT1 test. However, it should be remembered that comparison may seem difficult because previous studies did not all use the same criteria to define a positive ULNT. In this study, the Nee *et al*. criteria were used [24]. These authors insisted that the test should at least partially reproduce the patient’s symptoms and that they change with the SD maneuver. The same study concluded that the ROM difference of 10 degrees or more between the two sides in the elbow extension or completion of all movement sequences is not a valid criterion for defining a positive ULNT as in Wainner’s criteria used for several previous studies [16,27,28].

On the other hand, the reference standard used for this study is the same as in previous studies. Although it only explores large diameter fibers that are not always abnormal in the case of CTS [28], it is still recommended by the AEEM to detect positive cases of CTS because of its very high sensitivity and specificity [8]. It is also important to say that it measures a different dimension of neuropathy (Conduction velocity) than ULNTs (Mechansensitivity). Our sample size approaches that of Trillos *et al*. (2018) and is more important than the other studies [15,17,18]. Indeed, the clinical neurophysiology department of the RSH receives participants from different regions of Morocco and not only from Rabat, given the clinical expertise of its staff which could explain the size of the study sample. The other sample data (gender, age, chronicity of symptoms, and overuse of body movements) appear similar to those of other studies. The characteristics of our sample could reflect the epidemiology of CTS in the general population.

Our study findings concerning the ULNT2A accuracy corroborate those of Gracia *et al*. (2016) with their criterion B, which consists of the onset of symptoms in the wrist and the first three digits and change during SD, who estimated the sensitivity at 74%, specificity at 50%, a PLR of 1.47 and a NLR of 0.53. Wainner *et al*. in 2005 had a similar sensitivity of 75% but a lower specificity of 13% and NLR of 1.9 than the present study. On the other hand, the studies of Trillos *et al*. in 2018 and of Vanti *et al*. in 2011, which were based on Wainner's criteria to define a positive test, showed higher sensitivity, respectively, of 93% and 91.67%, but lower specificity than our study with 6.67% and 15% respectively. They have also estimated PLR at 1.04 and 1.07, while NLR was estimated at 1.00 and 0.55, respectively. However, the very liberal Wainner’s criteria could explain this increased sensitivity. Only a 10° difference in mobility of elbow extension between the two sides or reproduction of the patient's symptoms is sufficient to consider the test to be positive for Wainner's criteria. Thus, it appears that our study showed a good NLR compared to other studies [16,18,27] Table 3. We should emphasize that 92 participants (98%) in the present study had chronic CTS with symptoms lasting more than three months. So, our results have to be considered in symptomatic CTS with disease duration exceeding at least three months rather than in patients with potential CTS. On the other hand, it is a reality that CTS is more prevalent among women, and it is well documented in the literature. However, patient´s recruitment did not consider gender as a criterion for inclusion or exclusion. Thus, it should be mentioned that out of the total duration of the study, there were only two men who were excluded because of the exclusion criteria. Therefore, the results of this study can only be applied to women. Furthermore, it would have been desirable for the results of this study to be compared with other studies on the validity of ULNT2A in diagnosing CTS. Since such studies could not be found, the decision was made to compare them with studies concerning the validity of ULNT1 for the diagnosis of CTS, as the two tests are designated for the median nerve.

**Table 3:** the validity of ULNT1 and ULNT2A for diagnosing CTS

Study, year	Test	Sample size	Criteria	Sensitivity	Specificity	Positive LR	Negative LR
**Wainner RS *et al*. 2005**	ULNT1	n=82	Wainner´s criteria	75% (0.58-0.92)	13% (0.04-0.22)	0.86 (0.67-1.1)	1.9 (0.72-5.1)
**Vanti C *et al*. 2011**	ULNT1	n=47	Wainner´s criteria	91.67% (0.741-0.977)	15% (0.052-0.360)	1.0784 (0.377-3.083)	0.5556 (0.194-1.588)
**Vanti C *et al*. 2011**	ULNT1	n=47	Reproduction of symptoms 1^st^, 2^nd^ or 3^rd^ digit	54.17% (0.351-0.721)	70% (0.481-0.854)	1.8056 (1.132-2.879)	0.6548 (0.411-1.044)
**Vanti, C et al. 2012**	ULNT1	n=47	Reproduction of symptoms 1^st^, 2^nd^ or 3^rd^ digit	40% (0.256-0.564)	79.59% (0.664-0.885)	1.96 (1.275-3.012)	0.7538 (0.490-1.159)
**Bueno-Gracia E *et al*. 2016**	ULNT1	n=58	Reproduction of patient symptoms during the test and change with SD	58% (0.45-0.71)	84% (0.72-0.96)	3.67 (1.70-7.89)	0.5 (0.36-0.70)
**Bueno-Gracia E *et al*. 2016**	ULNT1	n=58	symptoms appear at the wrist or the first three digits of the affected hand and change during SD	74% (0.61-0.83)	50% (0.35-0.65)	1.47 (1.03-2.10)	0.53 (0.31-0.90)
**Trillos, MC *et al*.2018**	ULNT1	n=120	Wainner´s criteria	93% (88.21-96.79)	6.67% (0.0-33.59)	1 (0.90-1.10)	1.05 (0.25-4.89)
**Our study**	ULNT2A	n=94	Reproduction, at least partially, of the patient´s symptoms and change with SD	73.40% (0.62-0.84)	47.00% (0.17-0.78)	1.38 (1.12-1.65)	0.57 (0.21-1.15)

ULNT1: Upper Limb Neurodynamic Test 1 ; ULNT2A: Upper Limb Neurodynamic Test 2A; CTS: Carpal Tunnel Syndrome; SD: Structural Differentiation; (95% confidence interval)

## Conclusion

Because of the very low specificity of the test, the large uncertainties in the confidence intervals of sensitivity and likelihood ratios, the weak Kappa agreement, and the area under the curve value, the ULNT2A does not seem to have a value for the diagnosis of CTS in women when compared to NCS. However, it could help physical therapists, occupational therapists, and other concerned health care workers to detect an increased mechanosensitivity in the median nerve, especially in environments where access to NCS is limited.

### What is known about this topic

- The validity of ULNT2A for the diagnosis of carpal tunnel syndrome was not sufficiently studied;
- The prevalence of carpal tunnel syndrome is higher in women compared to men.
- Both ULNT1 and ULNT2A are designed to put mechanical stress on the median nerve and assess its mechanosensitivity.

### What this study adds

- The upper limb neurodynamic test 2A has a low to moderate value for diagnosing carpal tunnel syndrome in women;
- The diagnostic value of the upper limb neurodynamic test 2A is close to that of the upper limb neurodynamic test 1 when compared;
- The upper limb neurodynamic test 2A could represent an alternative to the upper limb neurodynamic test 1 in case of shoulder mobility restriction.
